# Study of variants associated with ventricular septal defects (VSDs) highlights the unique genetic structure of the Pakistani population

**DOI:** 10.1186/s13052-022-01323-5

**Published:** 2022-07-23

**Authors:** Sumbal Sarwar, Shabana, Amna Tahir, Zainab Liaqat, Saher Naseer, Rani Summeya Seme, Sabahat Mehmood, Saleem Ullah Shahid, Shahida Hasnain

**Affiliations:** Institute of Microbiology and Molecular Genetics, University of the Punjab, Lahore, 54590 Pakistan

**Keywords:** Ventricular septal defects, Pakistani population, Tetra ARMS PCR, RFLP, Genetic variations

## Abstract

**Background:**

Ventricular septal defects (VSDs) are one of the leading causes of death due to cardiac anomalies during the first months of life. The prevalence of VSD in neonates is reported up to 4%. Despite the remarkable progress in medication, treatment and surgical procedure for VSDs, the genetic etiology of VSDs is still in infancy because of the complex genetic and environmental interactions.

**Methods:**

Three hundred fifty subjects (200 VSD children and 150 healthy controls) were recruited from different pediatric cardiac units. Pediatric clinical and demographic data were collected. A total of six variants, rs1017 (ISL1), rs7240256 (NFATc1), rs36208048 (VEGF), variant of HEY2, rs11067075 (TBX5) and rs1801133 (MTHFR) genes were genotyped by tetra-ARMS PCR and PCR–RFLP methods.

**Results:**

The results showed that in cases, the rs1017 (g.16138A > T) variant in the ISL1 gene has an allele frequency of 0.42 and 0.58 respectively for the T and A alleles, and 0.75 and 0.25 respectively in the controls. The frequencies of the AA, TA and TT genotypes were, 52%, 11% and 37% in cases versus 21%, 8% and 71% respectively in the controls. For the NFATc1 variant rs7240256, minor allele frequency (MAF) was 0.43 in cases while 0.23 in controls. For the variant in the VEGF gene, genotype frequencies were 0% (A), 32% (CA) and 68% (CC) in cases and 0.0%, 33% and 67% respectively in controls. The allele frequency of C and A were 0.84 and 0.16 in cases and 0.83 and 0.17 respectively in controls. The TBX5 polymorphism rs11067075 (g.51682G > T) had an allelic frequency of 0.44 and 0.56 respectively for T and G alleles in cases, versus 0.26 and 0.74 in the controls. We did not detect the presence of the HEY2 gene variant (g.126117350A > C) in our pediatric cohort. For the rs1801133 (g.14783C > T) variant in the MTHFR gene, the genotype frequencies were 25% (CC), 62% (CT) and 13% (TT) in cases, versus 88%, 10% and 2% in controls. The ISL1, NFATc1, TBX5 and MTHFR variants were found to be in association with VSD in the Pakistani pediatric cohort whilst the VEGF and HEY2 variants were completely absent in our cohort.

**Conclusion:**

We propose that a wider programme of genetic screening of the Pakistani population for genetic markers in heart development genes would be helpful in reducing the risk of VSDs.

## Background

The most obvious cardiac abnormality of juveniles is ventricular septal defects (VSDs) as they encompass about 40% percent of all hereditary cardiac abnormalities in the form of an isolated defect. Ventricular septal defects are one of the leading causes of death in childhood during the first month of life [1]. The prevalence of neonatal VSD is reported up to 4% [2]. VSD can present as isolated heart disorder and as integral part of several other complex malformation including tetralogy of Fallot (TOF) and other syndromes [2]. VSD is classified into three categories on the basis of its genetic causes: VSD1 (*GATA4*), VSD2 (*CITED2*) and VSD3 (*NKX2.5*) [3]. Despite the remarkable progress in medication, treatment and surgical procedure for VSDs, the genetic etiology of VSDs is still in infancy due to complex genetic and environmental interaction. Certain transcription factors, termed pioneer transcription factors, play a significant role in initiation of cell programming and facilitating the cell differentiation and specification process. The *ISL1* gene is one of the important pioneer factors for cardiomyocyte differentiation. ISL1 is a LIM homeodomain transcription factor expressed transiently in the second heart field [4]. At the initial stages of heart developmental, *ISL1* serves as marker for progenitor cells. The human *ISL1* gene is located on chromosome 5q11.1 [5]. An *ISL1* gene knockout experiment in a mouse model has demonstrated the importance of this gene as knockout mice embryos died due to defects in heart development [6]. A SNP, rs1017 (NG_023040.1:g.16138A > T) in *ISL1* has been reported in association with congenital heart disease in humans [7]. NFATc1 (nuclear factor of activated T cells, cytoplasmic 1) is a transcription factor that belongs to the Rel family and is required for the development of cardiac valves [8]. Gene expression in pro-valve endocardial cells is determined by *NFATc1* via the activation of their heterogenic promoters [9]. The human *NFATc1* gene is located on chromosome 18q23. Studies have reported genetic mutations in *NFATc1* as the cause of atrio-ventricular septal defects (AVSD) [10]. A polymorphism rs7240256 (NG_029226.1:g.23449 T > C) in this gene has been reported as a risk factor of VSD. Vascular endothelial growth factor (*VEGF*) plays a key role in morphogenesis of the vascular system and differentiation of endothelial cells [11]. The human *VEGF* gene is located on chromosome 6p21.1. *VEGF* knockout experiments in mice have resulted in sudden death of 55% of animals. Even a single copy of the *VEGF* gene is not sufficient for the survival of mutant mice [12]. In humans, a polymorphism in the regulatory region of *VEGF*, rs36208048 (NG_008732.1:g.3877C > A) has been reported in association with VSD [13]. T-Box transcription factors (including TBX1, TBX2, TBX3, TBX5, TBX18 and TBX20) play a significant role in cardiac development such as endocardial formation and heart chamber maturation. So it is unsurprising that variants in such important transcription factors can lead to heart disorders [14]. *TBX5* is primarily known for cardiac forelimb development. It also acts as transcriptional activator for the genes specifically associated with cardiomyocyte maturation. The human *TBX5* gene is located on chromosome 12q24.21 [15]. Earlier studies has shown an association between the rs11067075 (NG_007373.1:g.51682G > T) in *TBX5* and VSDs [16, 17].

Hairy enhancer of split related with YRPW motif protein 2 (*Hey2* gene) belongs to a small family of HEY members consisting of basic helix loop helix (bHLH) transcription factors [18]. A key step in heart development is the timely and accurately addition of cardiac progenitor cells. HEY acts as regulator in the dynamics of cardiac progenitor cells [19]. The human *HEY2* gene is located on chromosome 6q22.31. Genetic variants in the *HEY2* gene cause heart defects [20]. A previous study reported a novel variant (NC_000006.10:g.126117350A > C) only in VSD patients [21]. *MTHFR*, a methylene tetrahydrofolate reductase, is an enzyme which catalyses synthesis of the initial circulatory form of folate, which in turn acts as a donor of methyl group for re-methylation of homocysteine to methionine [22]. The human *MTHFR* gene is located on chromosome 1p36.22. A common variant in *MTHFR*, rs1801133 (NG_013351.1:g.14783C > T), has been previously studied [23] and a recently published meta-analysis showed, an association of rs1801133 with the risk of heart disease [24]. Genetic variation in different ethnic groups is a confounding factor for human genetic disease research. Around the globe, genetic analysis has been performed to detect the relationship between a disease and the causative genes. In the same way, genetic research on VSD has also been conducted in different regions of the world. The aim of the present study was to determine the association of variants belonging to different genes including *ISL1*: rs1017 (NG_023040.1:g.16138A > T), *NFATc1*: rs7240256 (NG_029226.1:g.23449 T > C), *VEGF*: rs36208048 (NG_008732.1:g.3877C > A), *TBX5*: rs11067075 (NG_007373.1:g.51682G > T), *HEY2*: (NC_000006.10:g.126117350A > C) and *MTHFR*: rs1801133 (NG_013351.1:g.14783C > T), with VSD. The identification of different genetic polymorphisms in association with VSDs will aid surveillance and screening of VSD patients, facilitate genetic counselling and may help reduce the prevalence of this condition in the local region.

## Methods

### Ethical appsroval, written consent and recruitment of study subjects

The present study was approved by the local research ethical committee Institute of Microbiology and Molecular Genetics, University of the Punjab, Lahore Pakistan (approval # MMG1958-13/10/2020). Written informed consent was taken from the parents or guardians of patients. Two hundred samples (buccal swabs and blood) from isolated VSD patients were recruited from various pediatric cardiac wards of Lahore, Pakistan. One hundred and fifty healthy controls with no cardiac defects were included in this study. Sampling was done from 2018 to 2020.

### Inclusion/exclusion criteria

The patients diagnosed with isolated ventricular septal defects were selected via ECG. The image of the heart was captured by two dimensional and Doppler echocardiography to identify the magnitude of the shunt, the size and location of the defect (the whole structure of heart). During cardiac catheterization, the blood flow rate, the Qp/Qs ratio were also measured to determine the shunt size. Echocardiograms were performed by expert pediatric cardiologists. Cases and controls with syndromic VSD, other cardiac issues and infectious diseases (seropositive for HBV/HCV/HIV) were excluded.

### Data collection

Data from pediatric patients were collected on the basis of different parameters: hematological parameters like WBCs, RBCs, Hb level, platelets count, serum creatinine (SC), calcium, sodium, blood urea nitrogen (BUN), potassium, serum glutamic pyruvic transaminase, bilirubin, serum glutamic oxaloacetic transaminase, serum albumin, alkaline phosphate, and gamma GT etc., and demographic data like age, consanguineous marriage, family history, maternal Hb level during pregnancy. Use of medicine during pregnancy etc. was also recorded.

### Genotyping

The pediatric samples were taken in EDTA vials and preserved at -20 °C. Genomic DNA was extracted from the human leukocytes/WBCs. The quality of extracted DNA was evaluated using an Epoch Biotek micro-plate reader (Biotek Instruments, USA) and DNA concentration was adjusted to 10 ng/*u*L. The tetra primer ARMS PCR technique was used for the amplification of the *MTHFR*, *NFATc1* and *TBX5* genes (Table 1). For *HEY2*, *ISL1* and *VEGF* gene variants, the PCR–RFLP technique was used (Table 1). PCR reaction mixtures and conditions were optimized for each genetic marker. PCR and RFLP products were run on 2% agarose gel.

**Table 1:** Primer sequences, PCR product size, restriction enzymes and band sizes of SNPs selected for this study

SNPs	Gene	Primer sequence	PCR product size	Restriction Enzyme	Restriction Fragment Size
rs1017 NG_023040.1:g.16138A > T	*ISL1*	F-CTCTTGGCCTGTCCTGTAGC R-GCAATGCAAGAGCAAACAAA	318 bp	*DraI*	AT: 201, 117,95 and 22 bp AA: 201 and 117 bp TT:201, 95 and 22 bp
NC_000006.10:g.126117350A > C Asp98Ala (293A > C)	*HEY2*	F-GCCACAAGTACCCAGAAGAAAC R-GCACAAGTCTTCTCAACTCAG	340 bp	*MboI*	296 bp 44 bp
rs36208048 NG_008732.1:g.3877C > A	*VEGF*	F-AACCCCCATTTCTATTCAG R-CTGTGGAGTCTGGCAAAA	278 bp	AlwN1	Wild type cut at C 146 bp 132 bp
rs7240256 NG_029226.1:g.23449 T > C	*NFATc1*	FI- GGTCACATGCAGCAGCGCT RI- TGACATTTTCCACGCCTGACG FO-GGTTTGCAGTTAACCTTTTCCCA RO-AGAAAGCTCCTTCTGGCATAGG	–––	–––	196 bp (T allele wild type) 239 bp (C allele SNP) 384 bp outer primers
rs11067075 NG_007373.1:g.51682G > T	*TBX5*	FI- GTGATAAGGAATCAGCCGGGT RI- GAATCCCAACTGGAAGGAGAC FO- TCCTGCCTAGGAGACAACAAATA RO-AGACAATGAGGGGAAGTCAGATA	–––	–––	124 bp T allele 99 bp (wild type, G allele) 171 bp outer primers
rs1801133 NG_013351.1:g.14783C > T	*MTHFR*	OF-GCTGTTGGAAGGTGCAAGATCA OR-GAGTGGGGTGGAGGGAGCTTAT IF-AGAAGGTGTCTGCGGG IR-AAAGCTGCGTGATGAAAT	–––	–––	177 bp for T allele 230 for C allele 366 bp outer primer

### Statistical analysis

For the statistical analysis, the software SPSS version 22 was used. Genotypic frequencies of cases and controls were analysed using the chi-squared test (χ^2^) whereas allelic frequencies were calculated by direct counting. Genotypic and allelic frequencies were reported as percentages. Mean and standard deviation values were calculated for quantitative parameters. The study population was tested for Hardy–Weinberg equilibrium. As we analyzed 6 SNPs, the Bonferroni adjusted *p*-value (0.0125) was used as a threshold of significance for all analyses.

## Results

Patient mean age, family history of VSD, siblings with VSD, descriptive characteristics of hematological parameters, chemical profile and blood group distribution have been explained previously [25]. For the rs1017 (g.16138A > T) variant in *ISL1*, the genotype frequencies were of 52% (AA), 11% (TA) and 37% (TT) in cases in comparison to 21%, 8% and 71% respectively in controls (Table 2). The frequency of the T and A alleles is 0.42 and 0.58 respectively in cases, while 0.75 and 0.25 respectively, in controls (OR: 0.242, CI: 0.158–0.37, *p-value* < 0.0001). The genotype frequencies deviated from Hardy–Weinberg equilibrium for the cohort (*p-value*: < 0.0001). The genotypic distribution in dominant and recessive models is demonstrated in Table 3 (Dominant; OR: 0.21, CI: 0.11–0.39, *p-value*: < 0.0001, Recessive: OR: 0.24 CI: 0.13–0.43, *p-value*: < 0.0001, allelic OR: 0.242, CI: 0.157–0.37, *p-value*: < 0.0001) (Table 3).

**Table 2:** Genotypic frequencies in cases and controls

SNPs	Gene	Cases (*n* = 121)	Controls (*n* = 121)	*P* value
rs1017: NG_023040.1:g.16138A > T	*ISL1*	AA = 0.52 TA = 0.11 TT = 0.37	AA = 0.21 TA = 0.08 TT = 0.71	0.000215
rs36208048: NG_008732.1:g.3877C > A	*VEGF*	CC = 0.68 CA = 0.32 AA = 0.00	CC = 0.67 CA = 0.33 AA = 0.00	0.8921
rs7240256: NG_029226.1:g.23449 T > C	*NFATc1*	CC = 0.04 CT = 0.78 TT = 0.18	CC = 0.01 CT = 0.44 TT = 0.55	2.11 × 10^–5^
rs11067075 NG_007373.1:g.51682G > T	*TBX5*	GG = 0.21 GT = 0.70 TT = 0.09	GG = 0.56 GT = 0.36 TT = 0.08	1.6 × 10–4
NC_000006.10:g.126117350A > C(293A > C)	*HEY2*	AA = 1.0	AA = 1.0	––
rs1801133: NG_013351.1:g.14783C > T	*MTHFR*	CC = 0.25 CT = 0.62 TT = 0.13	CC = 0.88 CT = 0.10 TT = 0.02	2.09 × 10^–9^

**Table 3:** Dominant and recessive model analysis of the allelic frequencies of SNPs in this study

SNP (Gene)	Model	Genotype	Cases	Controls	OR (CI)	*P*-value
rs1017 (g.16138A > T) *(ISL1)*	Dominant	T/T	37 (36.6%)	71 (71%)	0.21 (0.11–0.39)	< 0.0001
		A/T-A/A	64 (63.4%)	29 (29%)	1.00	
	Recessive	T/T-A/T	48 (47.5%)	79 (79%)	0.24 (0.13–0.43)	< 0.0001
		A/A	53 (52.5%)	21 (21%)	1.00	
	**Alleles**	**T**	**85 (0.42)**	**150 (0.75)**	**0.242 (0.158–0.37)**	** < 0.0001**
		**A**	**117 (0.58)**	**50 (0.25)**		
rs7240256 (g.23449 T > C) *(NFATc1)*	Dominant	T/T	18 (17.8%)	55 (55%)	1.00	< 0.0001
		T/C–C/C	83 (82.2%)	45 (45%)	0.18 (0.09–0.34)	
	Recessive	T/T-T/C	97 (96%)	99 (99%)	1.00	0.16
		C/C	4 (4%)	1 (1%)	0.24 (0.03–2.23)	
	**Alleles**	**T**	**115(0.57)**	**154(0.77)**	**2.53(1.64–3.89)**	** < 0.0001**
		**C**	**87 (0.43)**	**46(0.23)**		
rs36208048 (g.3877C > A) *(VEGF)*	Dominant	C/C	69 (68.3%)	67 (67%)	1.00	0.84
		C/A	32 (31.7%)	33 (33%)	1.06 (0.59–1.92)	
	**Alleles**	**C**	**170 (0.84)**	**167 (0.84)**	**0.952(0.56–1.62)**	**0.8577**
		**A**	**32 (0.16)**	**33 (0.16)**		
rs11067075 (g.51682G > T) (*TBX5*)	Dominant	G/G	21 (20.8%)	56 (56%)	1.00	< 0.0001
		G/T-T/T	80 (79.2%)	44 (44%)	0.21 (0.11–0.38)	
	Recessive	G/G-G/T	92 (91.1%)	92 (92%)	1.00	0.82
		T/T	9 (8.9%)	8 (8%)	0.89 (0.33–2.40)	
	**Alleles**	**G**	**113(0.56)**	**148(0.74)**	**2.24(1.47–3.41)**	**0.0002**
		**T**	**89(0.44)**	**52(0.26)**		
rs1801133 (g.14783C > T) *(MTHFR)*	Dominant	C/C	25 (24.8%)	88 (88%)	1.00	< 0.0001
		C/T-T/T	76 (75.2%)	12 (12%)	0.04 (0.02–0.10)	
	Recessive	C/C–C/T	88 (87.1%)	98 (98%)	1.00	0.002
		T/T	13 (12.9%)	2 (2%)	0.14 (0.03–0.63)	
	**Alleles**	**C**	**113 (0.56)**	**186(0.93)**	**10.46(5.68–19.26)**	** < 0.0001**
		**T**	**89 (0.44)**	**14(0.07)**		

For the rs7240256 (g.23449 T > C) *NFATc1* variant the genotype frequencies were 4% (CC), 78% (TC) and 18% (TT) in cases compared to 1.0%, 44% and 55% in controls (Table 2). The minor allele frequency (MAF) was 0.43 in cases while 0.23 in the controls (OR: 2.53, CI: 1.64–3.89, *p-value* < 0.0001). In the dominant model, the OR was 0.18 with CI 0.09–0.34 and *p-value* < 0.0001 and in the recessive model OR was 0.24 with CI: 0.03–2.23 (*p-value*: 0.16) (Table 3). For the control group the genotype frequencies were in Hardy–Weinberg equilibrium (*p-value*: 0.021). Genotyping of rs36208048 (g.3877C > A), variant in the *VEGF* gene showed that the percentage of the AA, CA and CC genotypes were 0%, 32% and 68% respectively in cases versus 0%, 33% and 67% in controls (Table 2). The frequency of the C and A alleles were 0.84 and 0.16 respectively in cases and 0.83 and 0.17 in controls (*p-value* 0.8577). For the cohort, the genotype frequencies showed deviation from Hardy–Weinberg equilibrium (*p-value*: 0.066). For the dominant model the OR was 1.06 with CI: 0.59–1.92 (*p-value* 0.84). While recessive model analysis could not be performed due to absence of homozygous genotype in cases as well as in controls (Table 3).

For the polymorphism, rs11067075 (g.51682G > T) in *TBX5,* the frequencies of the GG, GT and TT genotypes were 21%, 70% and 9%, respectively in cases, versus 56%, 36% and 8% in controls (Table 2). The frequency of the T and G alleles was 0.44 and 0.56 respectively in cases, while 0.26 and 0.74 in controls (OR: 2.24, CI: 1.47–3.41 (*p-value* 0.0002). In the dominant model, the OR was 0.21 with CI 0.11–0.38 and *p-value* < 0.0001 versus in the recessive model, the OR was 0.89 with CI 0.33–2.40 and *p-value* 0.82 (Table 3). For the control group, the genotype frequencies were in Hardy–Weinberg equilibrium (*p-value*: 0.60). For the variant in *HEY2*: (g.126117350A > C), we did not detect the homozygous or the heterozygous genotypes. Therefore, dominant and recessive model analysis could not be performed in the cases or in controls. Dominant homozygous frequency was 1.0, both in cases and controls. The genotyping of rs1801133 (g.14783C > T) variant in *MTHFR* showed that the frequencies of the CC, CT and TT genotypes were 25%, 62% and 13%, respectively in cases, while 88%, 10% and 2% in controls (Table 2). The allelic frequency of T and C are 0.56 and 0.44 respectively, in cases, while 0.93 and 0.07 in controls (OR: 10.46, CI: 5.68–19.26, *p-value* (< 0.0001). In the dominant model, the OR was 0.04 with CI 0.02–0.10 (*p-value* < 0.0001) while in the recessive model, the OR was 0.14 with CI 0.33–0.63 and *p-value* 0.002 (Table 3). For the control group, the genotypic frequencies were in Hardy–Weinberg equilibrium (*p-value*: 0.65).

## Discussion

**Fig. 1:**
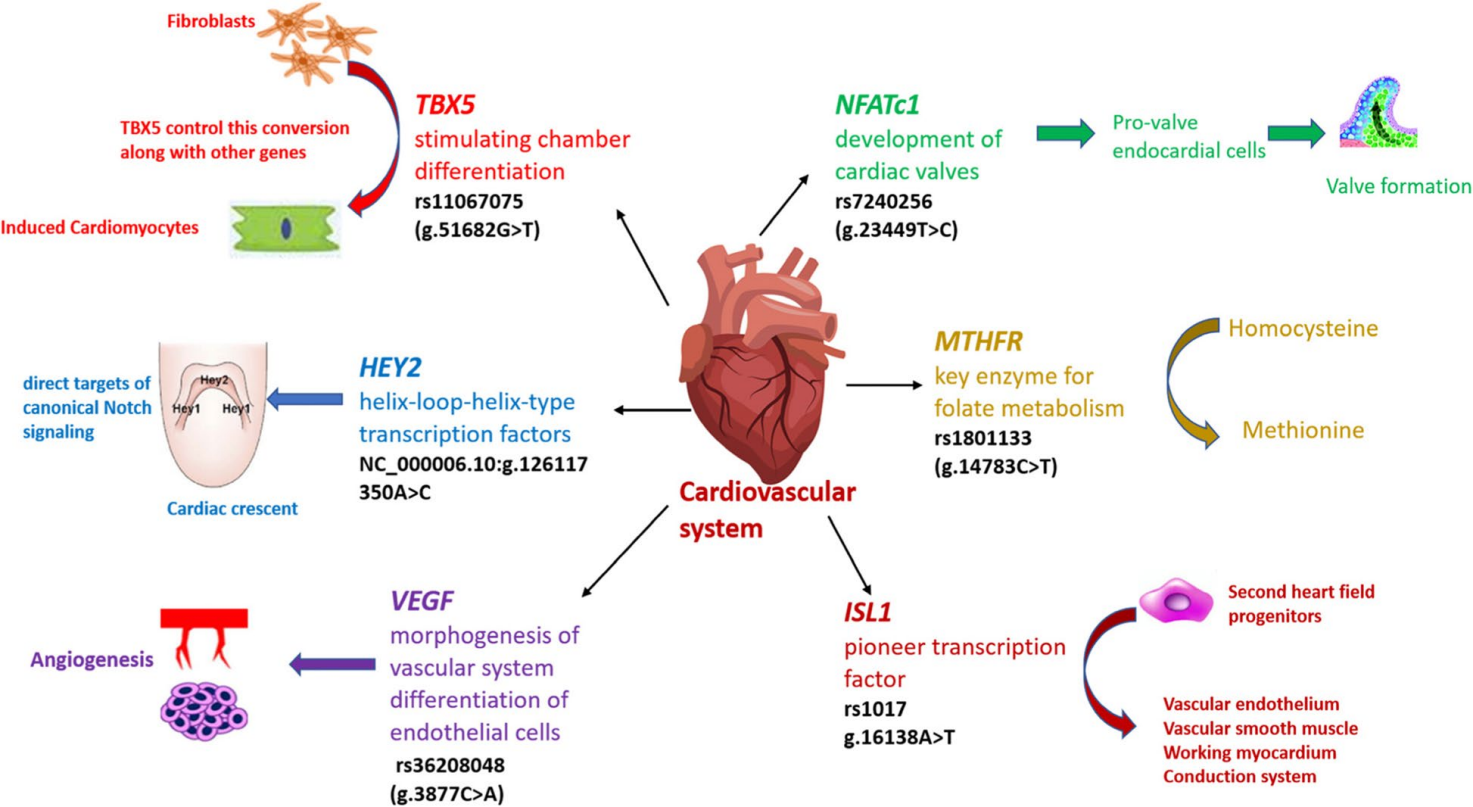
The role of the *ISL1, NFATc1, VEGF, HEY2, TBX5* and *MTHFR* genes in the cardiovascular system (SNPs selected for this study in black)

The healthcare burden of congenital heart disease is gradually increasing worldwide and more specifically in the low- and middle- income countries like Pakistan, where the cost of treatment is unaffordable for most families. Generally, the Pakistani population shows remarkable variations in the investigation of genetic diseases due to high rates of consanguineous marriages, a fact that makes this population attractive to geneticists. Advances in the field of genetics have led to the discovery of novel genetic markers for complex as well as for monogenic diseases [1]. Ventricular septal defect is the commonest congenital heart defect with 1 in 500 live births. Risk factors for VSD include genetic variations, family history, ethnicity and certain environmental factors. According to the data reported in previous studies, genes involved in the circulatory system and cardiac development such as, *GATA4, NKX2.5, TBX5*, *VEGF, MTRR, MTHFR, ISL1, NFATC1* and *CITED2* play crucial roles in the etiology of VSD [26]. Due to unavailability of pre- and post-natal biomarkers in clinical practice it is crucial to identify genetic risk or disease markers for VSDs. All genes (*ISL1, NFATc1, VEGF, HEY2*, *TBX5* and *MTHFR)* selected in this study are important structurally as well as functionally in cardiovascular system (Fig. 1).

The *ISL1* LIM-homeodomain transcription factor substantially contributes to embryonic heart development. It is one of the genes responsible for ventricle development [26]. Based on a previous study on a Chinese cohort, we selected rs1017 polymorphism as potential biomarker for prediction of VSD [27]. A study of meta-analysis suggested this polymorphism as a risk factor for heart disease [5]. In contrast to the Chinese population, no association was detected in a White population [28]. Surprisingly, in the current study, results showed the minor allele as the protective allele for VSDs in the Pakistani population. This was confirmed by genetic model analysis. Valvular and septal development is greatly affected by NFATc1. An earlier study reported significant association of variant homozygous genotype of rs7240256 with VSDs [29]. Our results are in consensus with a previous study where rs7240256 showed association with complications of VSDs [30]. Higher proportion of the risk genotype is an indicator of the effect of this polymorphism on valvular and septal development. VEGF is a signaling protein involved in angiogenesis. Any dysregulation in the function and structure of VEGF, can play a critical role in the pathogenesis of VSDs. Circulatory vasculature is under direct influence of growth factors like VEGF, any deviation from the normal function of this growth factor can lead to serious consequences [31]. From our findings, selected SNP rs36208048 of VEGF did not show statistical difference in allele frequencies between cases and controls despite this variant being located in the promoter region of gene which is an essential part for the normal function of the growth factor [13].

TBX5 transcription factors maintained their expression level throughout heart development and their persistent expression has also been observed in ventricular maturation [15, 32]. Our results are in concordance with a previous study, where variant rs11067075 in TBX5 was associated with VSD in pediatric patients [16]. In our study population, we observed different minor allele frequencies in cases and control with the risk allele playing a role in the association between variant and outcome. Dominant model analysis also supported these results. For the cardiac developmental pathways, HEY2 transcription factor plays an important role along with other factors like NKX2.5, TBX5 and GATA4 [33]. A study on a German population reported a novel polymorphism in the binding domain of HEY c.293A > C (p.Asp98Ala) (g.126117350A > C) [21]. Due to low sample size, sample bias and ethnic difference we did not find any significant difference in allelic and genotypic frequency in current cohort. However, this variation has been seen rarely, possibly due to its functionally important position in the HEY2 transcription factor. DNA, RNA and proteins need one carbon (methyl group) for methylation and re-methylation, usually catalysed by MTHFR. MTHFR also catalyses the conversion of homocysteine into methionine. Based on data published previously, the T allele of rs1801133 (g.14783C > T), is a risk factor for heart disorders [34–36]. Different ethnic groups including Egyptians, Tamilians, Iranian and Chinese have been reported to carry the T allele in association with congenital heart disease [22, 37–40]. Similarly, we obtained results in our cohort showing that the risk allele has a higher frequency in cases compared to controls. This suggests a significant association of the T allele with VSDs in the local ethnic groups.

## Conclusion

The Pakistani population is unique, in regard of its social, cultural, religious practices; it also has been reported to have a high percentage of consanguinity. This makes genetic research and in particular the study of complex genetic disorders including VSDs extremely challenging, but potentially rewarding. Although samples can be recruited from the entire country, their total number is limited due to lack of resources. This is the first attempt to determine the possible association of variants in the *ISL1, NFATc1, VEGF, HEY2*, *TBX5* and *MTHFR* genes with VSDs in a Pakistani cohort.

## Data Availability

All data is available with the corresponding author and can be accessed on request.

## Abbreviations

VSD: Ventricular septal defects
VEGF: Vascular endothelial growth factor
TBOX: T-Box transcription
ISL1: ISLET1
MTHFR: Methylene tetrahydrofolate reductase
SPSS: Statistical Package for Social Sciences
